# Efficacy of percutaneous microwave ablation guided by contrast-enhanced and two-dimensional ultrasound for in hepatic alveolar echinococcosis in difficult/dangerous locations

**DOI:** 10.3389/fmed.2024.1436753

**Published:** 2024-08-09

**Authors:** Wangxing Huang, Zhipeng Hu, Lina Qi, Xiaoyan Zhang, Min Li, Mingan Yu, Guoyong Hua

**Affiliations:** ^1^Graduate School of Qinghai University, Xining, China; ^2^Thoracic Surgery Department, Qinghai Provincial People’s Hospital, Xining, China; ^3^Interventional Ultrasound Department, Qinghai Provincial People's Hospital, Xining, China; ^4^Department of Interventional Medicine, China-Japan Friendship Hospital, Beijing, China

**Keywords:** alveolar echinococcosis, multilocular echinococcosis, hepatic alveolar echinococcosis, microwave ablation, contrast-enhanced and two-dimensional ultrasound

## Abstract

**Background:**

Ultrasound-guided microwave ablation (MWA) has become a popular method for treating malignant liver tumors. However, few studies have investigated its use in the treatment of hepatoalveolar echinococcosis (HAE). This study aimed to explore the effectiveness and safety of contrast-enhanced ultrasound combined with two-dimensional ultrasound-guided MWA for the treatment of HAE in difficult/dangerous locations.

**Methods:**

Data from 81 patients, who were diagnosed with hepatic alveolar hydatid disease in difficult/dangerous locations between January 2018 and January 2023, and underwent contrast-enhanced ultrasonography combined with two-dimensional ultrasound-guided MWA, were analyzed. After undergoing MWA, patients were followed up to determine whether the lesions recurred and to evaluate the therapeutic effect of MWA. Preoperatively, individualized strategies were designed for lesions in different locations, and different auxiliary ablation technologies were used for contrast-enhanced ultrasound combined with two-dimensional ultrasound-guided MWA to achieve complete inactivation of lesions in difficult/dangerous locations.

**Results:**

MWA was performed on 89 HAE lesions in 81 patients. The median diameter of the lesions was 2.86 cm (interquartile range [IQR] 2.36–3.49 cm). The complete ablation rate after surgery was 100%, with a recurrence rate of 11.11%, and median follow-up of 24 months (IQR 12–48 months). The incidence of minor complications was 14.81%; no serious complications or deaths occurred. Compared with before surgery, TB, DB, alanine aminotransferase, and aspartate aminotransferase levels increased (*p* < 0.001), albumin platelets and activated partial thromboplastin time decreased (*p* < 0.05), with no statistical difference in prothrombin time (*p* > 0.05).

**Conclusion:**

MWA may be a safe and effective method for treating HAE in difficult/dangerous locations, and may represent a new and alternative option for this patient population.

## 1 Introduction

Alveolar echinococcosis is a zoonotic disease caused by *Enterococcus multilocularis* larvae. Its growth pattern is similar to that of malignant tumors, with a 5-year mortality rate of 74%, and a 10-year mortality rate as high as 94% without treatment. The disease is also known as “insect cancer” (1). Treatment methods include radical resection, drug, and minimally invasive interventions. Drug therapy is mainly used for early stage lesions, while radical resection is the primary treatment option for advanced lesions. For larger lesions, radical resection has been adopted; however, some small lesions are at risk for progression after several years, and most smaller lesions grow in the first and second hepatic hilus. Some lesions are close to the hilar bile duct, the hepatic vein junction, and the top of the diaphragm; as such, complete surgical resection is virtually impossible during treatment (2). Currently, radical surgical resection combined with antiparasitic drugs is the first-line treatment for HAE (3). However, for small liver cancer lesions, radical resection involves more trauma and postoperative complications than microwave ablation (MWA) (4). MWA has yielded sound therapeutic effects in treating malignant liver tumors in difficult/dangerous locations by generating high temperatures around the lesion and causing coagulative necrosis in the lesion tissue. As such, MWA may be a promising treatment option for HAE in challenging locations. Few studies have investigated using MWA to treat HAE (5). Owing to insufficient early cases and short follow-ups, we mainly evaluated the efficacy of MWA in treating early HAE lesions in difficult/dangerous locations. The aim was to study the effectiveness and safety of contrast-enhanced ultrasound combined with two-dimensional ultrasound-guided MWA for treating HAE in difficult/dangerous locations.

## 2 Materials and methods

### 2.1 Patient selection

This study was approved by the Medical Ethics Committee of Qinghai Provincial People’s Hospital (Qinghai province, China) and conducted in accordance with the ethical guidelines of the Declaration of Helsinki. Due to the retrospective design of this study, requirements for informed written consent were waived. Clinical information from 81 patients diagnosed with difficult-site hepatoalveolar hydatids at the authors’ institution between January 2018 and January 2023, was collected. Ablation time and various auxiliary ablation techniques for patients with HAE in various difficult/dangerous anatomical locations were recorded, and timely follow-up was performed after surgery to analyze the effectiveness and safety of contrast-enhanced ultrasound combined with two-dimensional ultrasound-guided MWA.

### 2.2 Inclusion criteria

Inclusion criteria were as follows: (a) According to the 2010 World Health Organization expert consensus, HAE is diagnosed based on clinical manifestations, epidemiological data, imaging, serological indicators, histopathology and other items, (b) No sever coagulation disorders or hematological disorders.

### 2.3 Exclusion criteria

**Figure 1:**
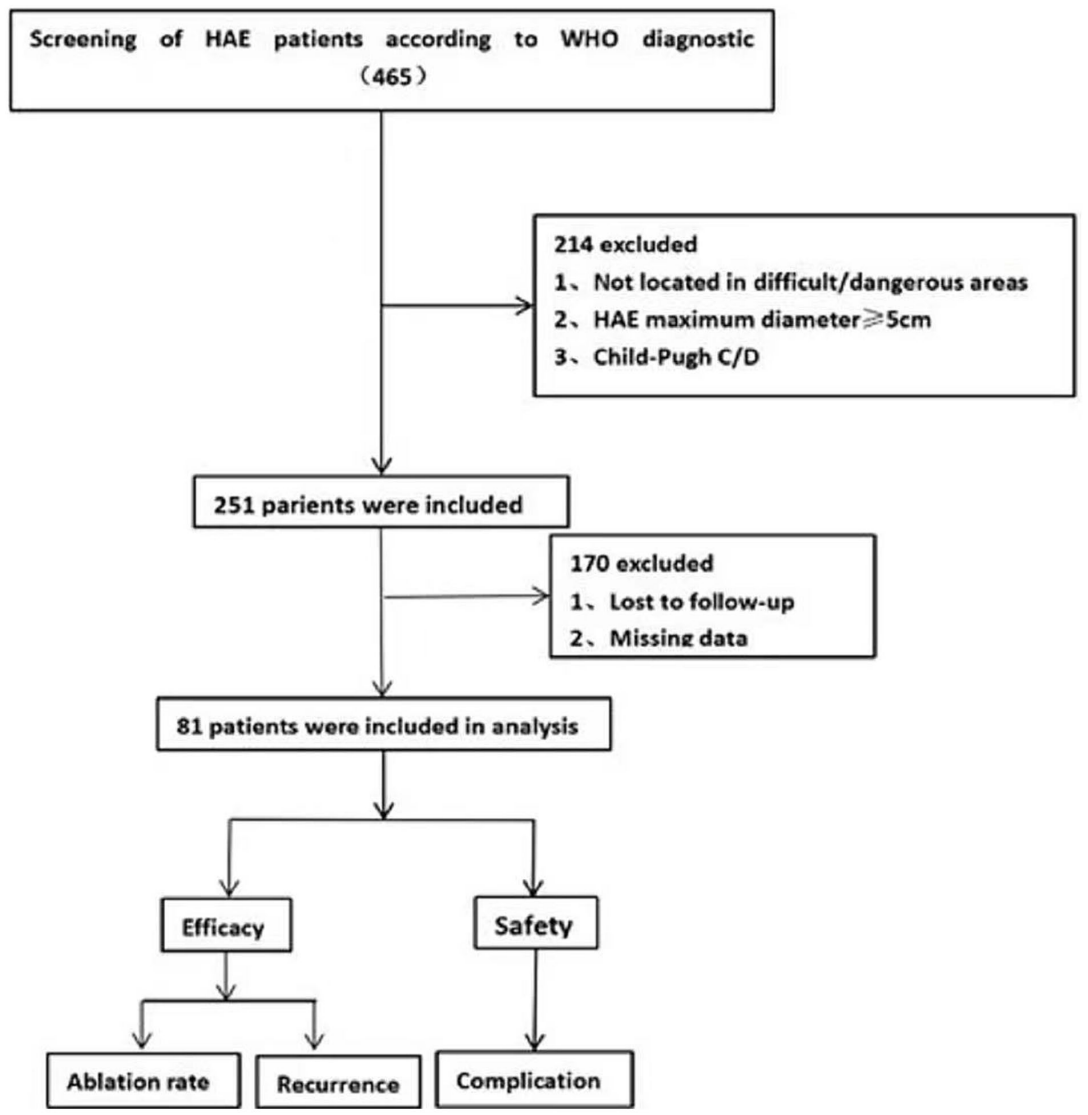
Flow-diagram illustrating the study process. Numbers enclosed in parentheses refer to the number of patients.

The exclusion criteria were: (a) Not located in difficult/dangerous areas, (b) HAE maximum diameter ≥ 5 cm, (c) Child-Pugh C/D, (d) Lost to follow-up, and (e) Missing data (Figure 1).

### 2.4 Contrast-enhanced ultrasound

**Figure 2:**
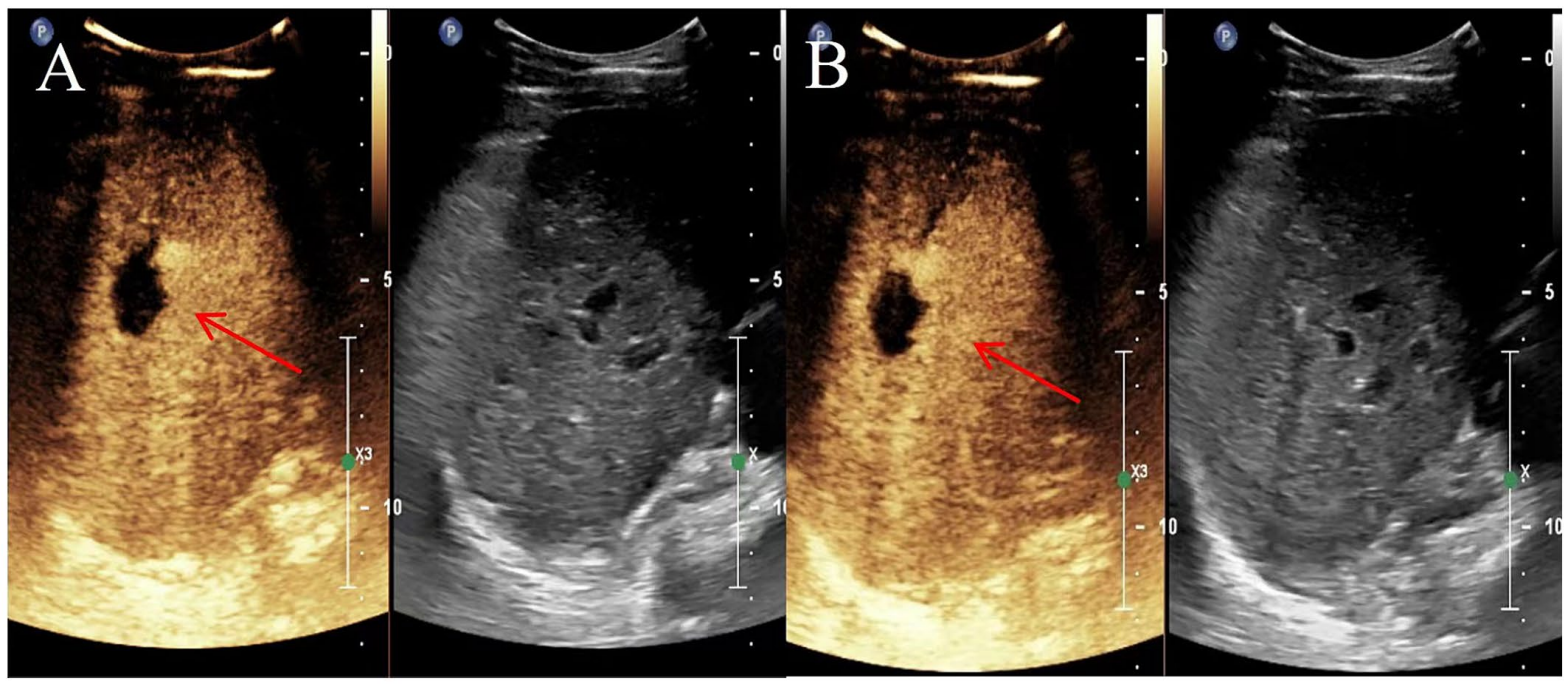
Representative images depicting lesions 6 and 12 months after microwave ablation (MWA) in a 40-year-old female with hepatoalveolar echinococcosis (HAE). **(A)** Contrast-enhanced ultrasound 6 months after ablation; **(B)** Contrast-enhanced ultrasound image 12 months after ablation. The red arrow indicates the lesion site.

All examinations were performed by three diagnosticians who performed liver contrast-enhanced ultrasonography. All patients fasted for 8 h before the examination. A routine ultrasound examination was performed first, followed by a contrast-enhanced ultrasound. Contrast agent (SonoVue, Bracco, Milan, Italy) was bolus-injected (2.4 mL) through the median cubital vein and then flushed with 12 mL normal saline (NS). The center, edge area, and surrounding liver tissue of the target lesion were observed continuously for 3 min. An analysis of 89 HAE lesions that underwent contrast-enhanced ultrasound examination revealed that the center of the lesion was non-enhancing in the arterial phase, portal venous phase and delayed phase, exhibiting “cavitation,” and the edge area gradually appeared from the arterial phase to the portal venous phase. Two-dimensional ultrasound combined with contrast-enhanced ultrasound before ablation can be a good diagnosis of hepatic alveolar echinococcosis, which is consistent with the results of enzyme-linked immunoassay. After ablation, the center and edge of the lesion were non-enhanced in the arterial phase, portal venous phase and delayed phase, exhibiting a “cavitation sign” (6) (Figure 2). Compared with conventional ultrasound, contrast-enhanced ultrasound can more accurately depict the blood supply range of the lesion edge area and determine the ablation margin (7). Contrast-enhanced ultrasonography after MWA can immediately verify the ablation effect and enable re-ablation of the incompletely ablated portion.

### 2.5 MWA procedure

An ultrasound device (EPIQ-5, Philips Healthcare, Best, Netherlands) equipped with an ultrasound probe (model C5-1) was used to examine and locate the puncture point, and all patients were administered intravenous anesthesia. Doppler ultrasonography was performed at the presumed puncture site to avoid large vessels in the path of the needle. The equipment included an MWA therapy device (KY-2000, Jiangsu Kangyou Medical Instrument Co., Ltd., Jiangsu, China) with an output power range of 1–100 W and an operating frequency of 2,450 MHz, and sterile disposable MWA needles. All lesions were ablated using an antenna by applying 20–40 W of microwave energy for 2.5–7.5 min each time. The ablation time, power, and auxiliary ablation parameters were determined based on the size and adjacency of the lesion, and an individualized ablation plan was developed for each patient. During treatment, routine ultrasonography was used to monitor the ablated hyperechoic areas to determine the treatment endpoints. After the lesion was ablated, the water-circulating MWA needle was slowly withdrawn, and the MWA needle channel continued to emit at a power of 80 W until the water-circulating MWA needle was pulled to the edge of the liver. This approach enables cauterization of the needle tract to prevent migration and minimize post-ablation bleeding. All procedures were performed by a physician with 10 years’ ablation experience, and the postoperative follow-up images were jointly analyzed by two physicians with 5 years’ experience in ultrasound diagnosis.

### 2.6 Clinical data collection

**Figure 3:**
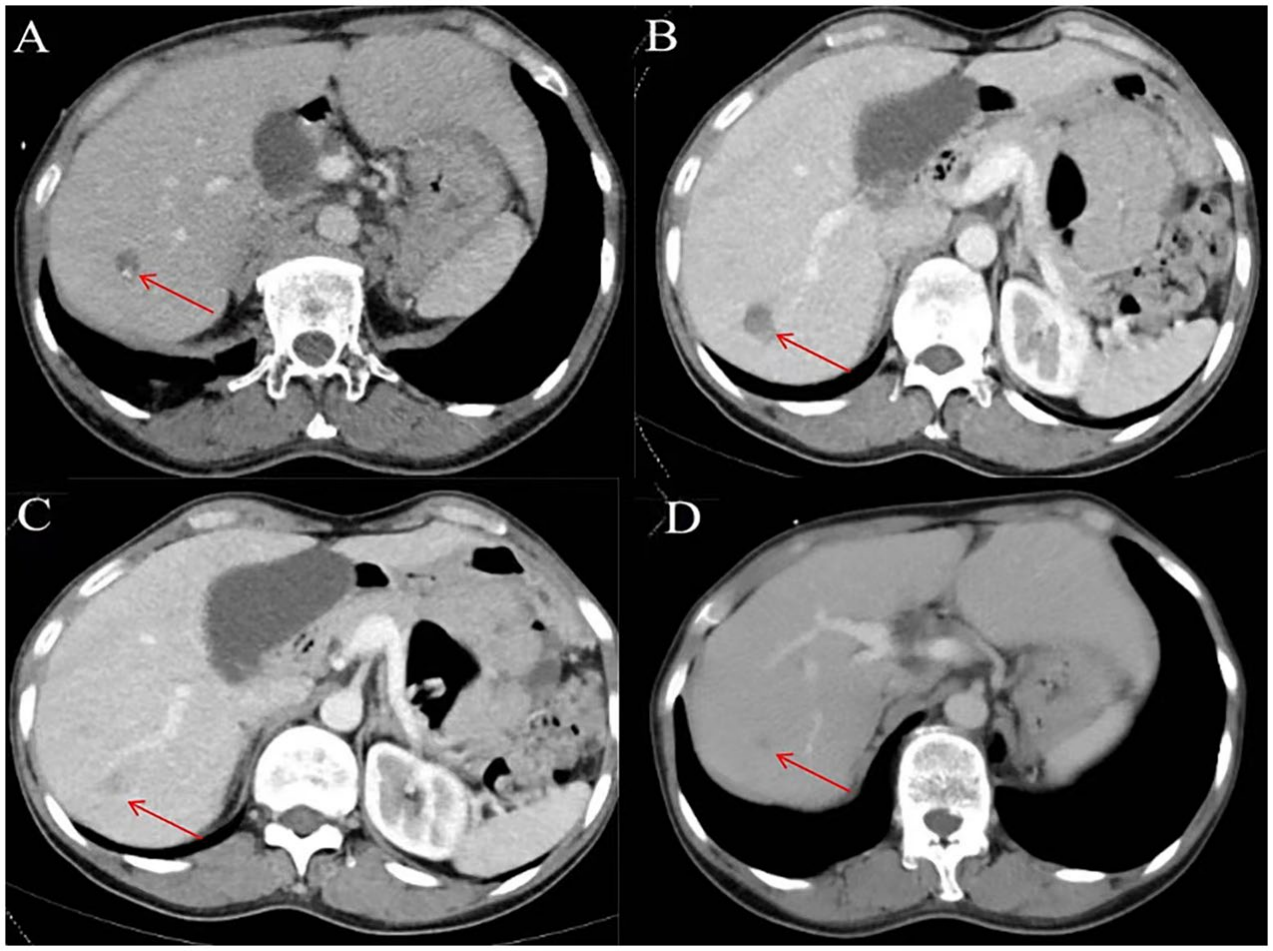
Representative images depicting lesions before and 1, 6, and 12 months after microwave ablation (MWA) in a 54-year-old female with hepatoalveolar echinococcosis (HAE). **(A)** Enhanced computed tomography (CT) performed before ablation; **(B)** Enhanced computed tomography (CT) 1 month after ablation; **(C)** Enhanced computed tomography (CT) 6 months after ablation; **(D)** Enhanced computed tomography (CT) 12 months after ablation. The red arrow indicates the HAE lesion.

Clinical information from all patients included demographic data (age, sex), symptoms, ablation-related laboratory investigation results, ablation-related information, and lesion imaging characteristics (size, location, and number). Efficacy evaluation and follow-up included contrast-enhanced ultrasound examination within 24 h after ablation. Complete ablation was defined as ablation without vascular contrast-agent filling (6). CT or ultrasound should be performed for the first month after surgery and every six months after surgery (Figure 3). Peripheral recurrence were defined as those with imaging features of hepatic hydatid around the ablation area, and distant metastases were defined as imaging features of hydatid outside the liver.

### 2.7 Statistical analysis

Statistical analysis was performed using SPSS version 25.0 (IBM Corporation, Armonk, NY, USA) for Windows (Microsoft Corporation, Redmond, WA, USA). The Shapiro–Wilk test was used to determine whether the continuous variables were normally distributed. Normally distributed continuous variables are expressed as mean ± standard deviation (SD) and analyzed using sampling tests. Continuous variables with non-normal distributions are expressed as mid-and four-quartile distributions (interquartile range [IQR]) and analyzed using the Wilcoxon test. Differences with *p* < 0.05 were considered to be statically significant.

## 3 Results

### 3.1 Demographic and clinical characteristics

This study retrospectively analyzed the clinical data, relevant demographic indicators, MWA materials, and clinical complication rates in 81 patients diagnosed with hepatic alveolar hydatid disease in difficult locations and underwent MWA. Baseline clinical characteristics, lesion-related information, and MWA performed in the 81 patients (42 male, 39 female; mean [± SD] age, 33.64 ± 10.06 years) are summarized in Table 1. Fifty-eight percent of patients had clinical symptoms, and 77% were Child–Pugh class A. All lesions were <5 cm, 72.83% of patients had lesions <3 cm in diameter, and 27.17% of patients had lesions between 3 and 5 cm in diameter. Of the patients, 88.88% had solitary lesions, 8.64% had 2 lesions, and 2.48% had 3. The lesions were more common in segments III (27.16%) and VI (20.98%), and in segments V (14.81%), VII (12.34%), and IV (11.11%). The lesions were located in the right and left liver in 55.60 and 44.40% of cases, respectively. No recurrence was observed in 72 patients after surgery, and the lesions gradually stabilized. Among these, 49 had calcification bands at the edges of the lesions during follow-up, and no expansion of the lesions was found during multiple follow-up visits. In 23 patients, the lesions gradually shrank during postoperative follow-up until they became stable. The ablation protocols and clinical results are summarized in Tables 2–4.

**Table 1:** Patient characteristics.

Characteristic	Value
Mean age (year)^a^	33.64 ± 10.06
Sex	
Male	42 (51.85)
Female	39 (48.15)
Ethnicity	
Han	3 (3.70)
Tibetan	78 (96.30)
Hepatitis B	
Yes	10 (12.35)
No	71 (87.65)
Clinical symptom	
Yes	47 (58)
No	34 (42)
Child–Pugh grade	
Child A	77 (95)
Child B	4 (5)
Maximum diameter of nodules (cm)	
<3 cm	59 (72.83)
3–5 cm	22 (27.17)
Number of nodules	
1	72 (88.88)
2	7 (8.64)
3	2 (2.48)
Segmental location	
I	1 (1.23)
II	8 (9.87)
III	22 (27.16)
IV	9 (11.11)
V	12 (14.81)
VI	17 (20.98)
VII	10 (12.34)
VIII	2 (2.50)
Right hepatic lobe	55.60%
Left hepatic lobe	44.40%

Except where indicated, data are raw data, with percentages in parentheses.

^a^Data are means ± standard deviation.

**Table 2:** The laboratory examinations before and after MWA.

Laboratory test	Before MWA	After MWA	*p* value
ALB (g/L)^a^	41.05 (39.10–42.10)	40.20 (39.10–40.90)	0.001
TB (μmol/L)^a^	12.50 (11.60–13.20)	22.30 (19.32–23.82)	0.000
DB (μmol/L)^a^	2.50 (2.10–2.80)	3.40 (2.80–3.90)	0.000
PLT (109/L)^a^	249.00 (240.00–263.00)	218.50 (206.25–226.00)	0.000
ALT (U/L)^a^	23.00 (16.00–31.00)	144.00 (120.50–165.00)	0.000
AST (U/L)^a^	22.50 (18.00–27.00)	91.50 (80.00–112.00)	0.000
PT (s)^a^	11.60 (10.92–12.40)	11.50 (10.90–12.07)	0.568
APTT (s)^a^	28.50 (26.30–32.27)	27.50 (26.90–28.60)	0.000

*p* < 0.05 was considered statistically significant.

^a^Data are median, with IQR in parentheses, analyzed using the paired Wilcoxon-test.

**Table 3:** Strategies for needle placement depending on HAE lesion.

Location	Strategies	Value
Near the diaphragm	Transhepatic access with angled intercostal approach, artificial pleural effusion-assisted ablation	38.20% (34/89)
Near large vessels	Needle path parallel to the blood vessel as possible avoiding direct puncture, using intermittent ablation with different powers	28.09% (25/89)
Near the important organs	Insert the needle parallel to or away from important organs, and artificial ascites assists ablation	33.71% (30/89)

**Table 4:** Ablation protocol and clinical results.

Ablation protocol	Value
Complete ablation for the first time	100 (89/89)
Ablation power^b^	30 (20–40)
Ablation time^b^	4 (4–4.25)
Follow-up time^b^	24 (12–48)
Recurrence rate	11.11 (9/81)

Except where indicated, data are raw data, with percentages in parentheses.

^b^Data are median, with IQR in parentheses.

### 3.2 Evaluation of the effectiveness of MWA

Imaging results within 24 h after surgery revealed that 100% (89/89) of the lesions were completely ablated. After MWA, patient symptoms improved before discharge. In addition, as of January 31, 2023, the median follow-up duration was 24 months (IQR 12–48 months). During follow-up, 11.11% (9/81) of patients developed intrahepatic recurrence of HAE, with a median recurrence time of 19 months. No deaths occurred due to extrahepatic metastases.

### 3.3 Complications

According to the Clavien-Dindo classification system (7), there were no serious complications or deaths in the 81 patients; postoperatively, 7 grade 1 complications and 5 grade 2 complications were recorded, all of which were minor. Three patients with lesion diameters of 4.63 cm, 4.23 cm, and 4.21 cm, respectively, developed post-ablation syndrome (PAS), which is low-grade fever caused by the release of inflammatory factors due to tissue necrosis caused by thermal ablation, and only required supportive treatment (8). Four patients with lesion diameters of 3.71 cm, 3.46 cm, 3.26 cm, and 2.63 cm developed postoperative hypoalbuminemia and only needed albumin supplementation. Four patients with lesion diameters of 3.96 cm, 3.65 cm, 3.54 cm, and 2.86 cm at the top of the diaphragm developed postoperative asymptomatic pleural effusion, which required only observation and no special treatment, such as ultrasound-guided pleural effusion puncture and drainage. In one patient, a lesion diameter of 3.36 cm located at the attachment of a large blood vessel caused transient jaundice after ablation. After the administration of anti-jaundice, jaundice disappeared within 2 days (Table 5).

**Table 5:** Post MWA complications.

Complications	Location	Value
Minor (I–II)		14.81% (12/81)
Post-ablation syndrome	Adjacent to gastrointestinal tract, near the diaphragm	3.70% (3/81)
Hypoproteinemia	Near large vessels, adjacent to gastrointestinal tract, near the diaphragm	4.94% (4/81)
Asymptomatic pleural effusions	Near the diaphragm	4.94% (4/81)
Jaundice not requiring therapy	Near large vessels	1.23% (1/81)

## 4 Discussion

MWA is a first-line treatment for small liver cancers. Previous studies have demonstrated that MWA can be used to treat alveolar hydatids. We retrospectively analyzed clinical data from 81 patients with liver alveolar hydatids in difficult locations. Results of the present study demonstrate that MWA can be used to treat alveolar hydatids. Ablation is used in patients with liver alveolar hydatids located in difficult or dangerous locations. It has a shorter operative duration, fewer complications, shorter postoperative hospitalization, and a low recurrence rate. Finally, we believe that MWA is an effective and safe method for treating alveolar hydatids in difficult or dangerous locations.

In our study, contrast-enhanced ultrasound examination was performed before and after surgery to more accurately select the edge area of hydatid lesions and guide the coverage of the edge ablation zone. All lesions were located in difficult/dangerous locations in the 81 patients included in this study. The general ablation method used was difficult to perform completely and was prone to complications. Therefore, HAE lesions located on the top of the diaphragm are hidden due to their proximity to the diaphragm, had limited surgical field of view, and variable temporal position of the lesions in the respiratory cycle (9). Preoperatively, 0.9% sodium chloride (i.e., normal saline [NS]) injection was used to establish suitable artificial pleural effusion to keep the puncture field clearly identifiable and to reduce motion artifacts by reducing the ventilation tidal volume and the Valsalva maneuver (10). It then passes through the right lower chest wall and reaches the lesion at the top of the right diaphragm. Microwave radiation is used to ablate lesions while protecting the lungs. During ablation, the MWA needle passes through the pleura, which may cause pneumothorax. To avoid this problem, we used artificial pleural effusion and real-time ultrasound guidance (11). For lesions located around the intrahepatic blood vessels and bile ducts, ablation using fixed power is more likely to cause complications. As such, 40 W of ablation power was used at the distal end of the lesion, and a lower power (20 W) was selected at the end close to the blood vessels and bile ducts, using an ablation time of 10 s. A water circulation MWA needle was deployed parallel to the bile duct to reduce damage to the adjacent bile ducts (12). For lesions adjacent to the surrounding bowel, direct ablation increases the risk for residual disease and local progression (13). Additionally, complications, such as abdominal bleeding and gastrointestinal injury, are likely to occur in the liver adjacent to the intestine (14). For this part of the HAE lesion, we used hydrodissection-assisted ablation and preoperatively placed a needle between the liver capsule and the adjacent structures (such as the gastrointestinal tract, gallbladder, and upper pole of the kidney) under ultrasound guidance (15). Once the needle tip was in place, the needle core was removed, and 0.9% NS was injected through the catheter connected to the puncture needle. If there was no resistance to the injection and the NS was not localized during imaging, the catheter was connected to an NS bag (11). A total of 300–1,500 mL NS was infused until at least a 0.5 cm separated liver capsule was observed at the hepatic hydatid lesion site from adjacent key structures under two-dimensional ultrasound (14).

In a previous study, Li et al. (11) reported that a total of 91 (94.8%) of 96 subcapsular tumors were ablated, and 18.8% (18/96) of local tumors progressed. At the same time, no major complications were reported, and minor complications (minor pain and asymptomatic pleural effusion) did not require specific measures. In addition, Huang et al. (16) used MWA to treat 163 perivascular lesions, with successful initial ablation in 157 (96.3%) and local tumor progression detected in 22 (13.5%). Makovich et al. (17) used MWA to treat hepatocellular carcinoma at the top of the diaphragm in 38 patients. The incidences of complications and local progression after ablation of lesions on top of the diaphragm were similar to those after ablation of hepatocellular carcinoma in ordinary locations. There was no statistical difference in the incidence or local progression rates of the disease. The complete ablation rate in the present study was 100%. Although the recurrence rate was 11.11% (9/81 patients), the results were acceptable. Deng et al. (18) reported a recurrence rate of hepatic alveolar hydatid lesions after MWA in common locations of 13% (6/45), which was higher than the recurrence rate in the present study. Mukund et al. (19) reported that the technical effectiveness of MWA for hepatocellular carcinoma in difficult locations was 83%. The technical effectiveness rate in our study was 85% (80/89), which is greater than previous research results. In our study, there were no serious complications or deaths among the 81 patients, and all complications were minor, with an incidence rate of 14.81% (12/81). Deng et al. (18) in the MWA of hepatic alveolar hydatid. Clinical studies have pointed out that postoperative complications of MWA of hepatic alveolar hydatids in common locations are all minor, with an incidence rate of 11.1%, which is similar with our research results. In radical surgery for alveolar hydatid, the key to determining cure is complete resection of the lesion edge (20). Our study also found that the key to cure with MWA lies in coverage of the lesion edge. This is believed to be due to the presence of an infiltrative zone around HAE lesions, which have growth characteristics similar to those of malignant tumors and invade surrounding normal tissues. The main reason for HAE recurrence is incomplete inactivation of the marginal infiltration zone (21). Because it is difficult to accurately determine the scope of the infiltration area using two-dimensional ultrasound, in our study, real-time contrast-enhanced ultrasound was used to define the scope of the infiltration area before surgery. The range determined by contrast-enhanced ultrasound is usually larger than that of the lesion determined by two-dimensional ultrasound. The average power we used during lesion ablation was 31.23 W. In addition to the ablation power, the occurrence of complications may also be related to the frequency of the microwave device. Currently, there are two main frequencies for microwave ablation: 915 MHz and 2,450 MHz. The frequency used in this study was 2,450 MHz. Sun et al. (22) reported that a frequency of 915 MHz was more likely to produce deeper penetration than 2,450 MHz. Therefore, the ablation area produced by high-frequency microwaves is easier to control and the incidence of complications is lower. Discomfort and low-grade fever after ablation are considered manifestations of PAS, which may be the result of an inflammatory response and cytokine production in the necrotic tissue after ablation. Among them, lesions <3.25 cm in diameter were less likely to lead to PAS after ablation (23), which is consistent with our research results. These patients only needed supportive care. Postoperatively, four patients developed postoperative hypoalbuminemia. No special treatment was required, and albumin supplementation was sufficient. Four of the 81 patients developed asymptomatic pleural effusion, and the occurrence of pleural effusion after thermal ablation was transient pleurisy related to the thermal effect. Direct thermal injury to the pleura may result in increased pleural capillary filtration and interference with parietal pleural fluid clearance, leading to the formation of pleural effusion (24). One of the 81 patients had a lesion diameter of 2.56 cm and was located at the attachment of a large blood vessel. The patient developed mild jaundice postoperatively. Two-dimensional ultrasonography revealed no evident bile duct damage. Jaundice resolved spontaneously after administration of anti-jaundice. In our study, all complications were minor and there were no deaths. Furthermore, these complications were self-limiting and required no specific intervention. Although transaminase levels increased after ablation in our study, this only required supportive care. Andreano et al. (25) speculated that the total amount of ablation was related to an increase in transaminase levels after surgery.

Finally, Qinghai Provincial People’s Hospital treats about 40 cases of HAE with ultrasound-guided MWA every year, and about 20 patients are located in difficult/dangerous locations. The present study had some limitations. First, this was a retrospective study without a control group; however, we compared our results with those of other researchers. Second, all lesions in our study were <5 cm in diameter, and there was a lack of data supporting ablation in patients with lesions >5 cm. Third, this was a single-center study with insufficient sample size and a large selection bias.

## 5 Conclusion

Results of the present study indicated that contrast-enhanced ultrasound combined with two-dimensional ultrasound-guided MWA is a safe and effective method for the treatment of hepatic alveolar hydatids in difficult/dangerous locations. This provides a new treatment method for patients with liver alveolar hydatids in whom surgery is not possible.

## Data availability statement

The original contributions presented in the study are included in the article/supplementary material, further inquiries can be directed to the corresponding authors.

## Ethics statement

The studies involving humans were approved by the Scientific Research Ethics Committee of Qinghai Provincial People’s Hospital. The studies were conducted in accordance with the local legislation and institutional requirements. The Ethics Committee/Institutional Review Board waived the requirement of written informed consent for participation from the participants or the participants’ legal guardians/next of kin because this study is retrospective and has been approved by the Ethics Committee to exempt informed consent.

## Author contributions

WH: Data curation, Writing – original draft, Writing – review & editing. ZH: Data curation, Writing – review & editing. LQ: Data curation, Writing – review & editing. XZ: Data curation, Writing – review & editing. ML: Data curation, Writing – review & editing. MY: Data curation, Funding acquisition, Methodology, Writing – review & editing. GH: Data curation, Formal analysis, Funding acquisition, Methodology, Writing – original draft, Writing – review & editing.
